# DNA Damage: Health and Longevity

**DOI:** 10.1155/2018/9701647

**Published:** 2018-09-13

**Authors:** Sharbel Weidner Maluf, Wilner Martínez-López, Juliana da Silva

**Affiliations:** ^1^Laboratory of Genetics, University Hospital, Universidade Federal de Santa Catarina, Florianópolis, SC, Brazil; ^2^Epigenetics and Genomic Instability Laboratory, Instituto de Investigaciones Biológicas Clemente Estable, Montevideo, Uruguay; ^3^Laboratory of Genetic Toxicology, PPGBioSaúde and PPGGTA, Lutheran University of Brazil (ULBRA), Canoas, RS, Brazil

The process of aging results in a host of changes at the cellular and molecular levels, which include genomic instability. Over the entire life course, these changes can be seen in unhealthy manifestations, especially in advanced age with its associated functional declines caused by the accumulation of cellular damage, together a diminished ability to repair this damage. Most of the time, the results of DNA damage include malformations, cancer, aging, and cell death.

The maintenance of genomic stability has been considered, in several studies, as the main factor that leads to human longevity. Information that can be extracted from older populations that have achieved greater longevity is a valuable source of knowledge that can be used to break down this complicated network of cellular responses that leads to aging. Y. J. Kim et al. used genomic database of oldest-old population to understand the association of DNA repair with longevity in a very interesting review.

Cell homeostasis is the result of a network of biochemical factors; failure to maintain this homeostatic balance may occur with aging. In addition to endogenous factors, environmental exposure to stressors can lead to damage accumulation and disease susceptibility. It is important to point out that a healthy environment is critical to ensuring healthy human populations. Telomeres play a critical role in protecting chromosomal end fusions, degradation, abnormal recombination, and other detrimental chromosomal events that lead to increased genomic instability and aging. Optimum telomere function and length are important for cell proliferation and apoptosis. Critically short telomeres initiate senescence resulting either in apoptosis or cell cycle arrest. Other phenomenon associated with aging is a change in patterns of epigenetic modifications. Epigenetics is most commonly defined as modifications to DNA and DNA packaging that do not involve changes to the DNA sequence and that are potentially transmissible to daughter cells.

V. F. S. Kahl et al. evaluated the effect of dietary intake and genetic susceptibility polymorphisms in genes on genomic and epigenetic instability in individuals exposed to pesticides. They showed increased levels of different parameters of DNA damage, including reduction of telomere length. Global DNA methylation was also decreased in farmers. The influence of diet is also discussed in this study, which suggests an important role of dietary intake and subjects' genetic susceptibility to xenobiotic-induced damages and epigenetic alterations in tobacco farmers occupationally exposed to mixtures of pesticides. Susceptibility is critical to an understanding of environmental diseases, including cancer, and many xenobiotic agents act to alter susceptibility. Unknown individual susceptibility, inadequate toxicity data, and the unpredictable nature of interaction effects make the implementation of a human biomonitoring assessment for complex mixtures of chemicals extremely complicated.

M. Wezyk et al. discuss that epigenetic mechanisms play an important role in the development and progression of various neurodegenerative diseases, including Alzheimer's disease. The study reinforces the view that the genetic methylation status in the blood may be a valuable predictor of molecular processes occurring in affected tissues.

T. Moriwaki et al. have shown that ATM (mutated ataxia-telangiectasia) induces cell death with autophagy in nondividing cells of *Caenorhabditis elegans* in response to exposure to H_2_O_2_. ATM kinase is a master regulator of the DNA damage response and is directly activated by ROS in addition to DNA double-stranded breaks.

T. R. D. Hamilton et al. discuss that the most accepted causes of sperm DNA damage are deleterious actions of ROS, defects in protamination, and apoptosis, as well as sperm DNA fragmentation is considered as one of the main causes of male infertility. They evaluated the effects of heat stress on the chromatin of ejaculated and epididymal sperm and the activation of apoptotic pathways in different cell types in ram testis. The study demonstrated that testicular heat stress increases ram sperm DNA fragmentation without changes in protamination and apoptotic patterns.

R. S. Fortunato et al. show that the H_2_O_2_-generating enzyme DUOX1 plays an important role in maintaining genomic stability, demonstrating that this enzyme is silenced in breast cancer, using shRNA to knock down its expression in nontumor cells (MCF12A).

Carcinogenesis is directly related to the prolonged accumulation of injuries at different biological levels, which alter the cells both genetically and biochemically. In each of these situations, there is an opportunity for intervention—a chance to prevent, delay, or stop the gradual march of healthy cells towards malignancy. A new strategy to reduce its incidence relates to intervention programs for diet and nutrition, as well as for the development of pharmacological products that could work as chemopreventives. Nonenzymatic antioxidants such as ascorbate, tocopherols, carotenoids, and flavonoids in general, present in diets rich in fruits and vegetables, are important defenses against free radicals, reducing the chances of developing degenerative pathologies.

M. F. C. J. Paz et al. showed persistent increased frequency of genomic instability in women diagnosed with breast cancer. The variability of the DNA damage of breast cancer patients that can be related to diet, from the diagnosis until the end of the oncological treatment, demonstrated more susceptible to oxidative stress. They concluded that early diagnosis indicates a good prognosis and is fundamental in patient survival, being able to signal a less aggressive treatment.

Alkylating agents (AAs) used as chemotherapy are able to induce alkylation in macromolecules, causing DNA damage, as DNA methylation. C. F. Araujo-Lima et al. evaluate atorvastatin (AVA) antimutagenic, cytoprotective, and antigenotoxic potentials against DNA lesions caused by AA. This study supports the hypothesis that statins can be chemopreventive agents, acting as antimutagenic, antigenotoxic, and cytoprotective components, specifically against alkylating agents of DNA.

A decline in the efficiency of mitochondrial action with age would cause release of higher concentrations of reactive oxygen. This would be exacerbated by a decline in the effectiveness of antioxidant defenses or DNA repair pathways. Recent animal studies have shown that mitochondrial dysfunction initiates and accelerates renal injury in sepsis. Q. Hu et al. evaluated the efficiency of urinary mitochondrial DNA (UmtDNA) as a marker of renal dysfunction during sepsis-induced acute kidney injury (AKI) and suggested that UmtDNA may be regarded as a valuable biomarker for the occurrence of AKI and the development of mitochondria-targeted therapies following sepsis-induced AKI.

Damage to human genomic DNA occurs very frequently, and the vast majority of these damages are successfully repaired by mechanisms involving many biochemical factors that characterize the DNA damage response (DDR). One theory of aging proposes that it results from the accumulation of oxidative damage caused by the action of free radicals (reactive oxygen species, ROS) on macromolecules such as DNA, proteins, and lipids, leading to a loss of function of these molecules. Many studies suggest that ROS participate in the pathophysiological mechanism of various human diseases, including Parkinson's disease, multiple sclerosis, muscular dystrophy, cataracts, retinopathies, atherosclerosis, myocardial infarction, ischemia and reperfusion syndrome, pulmonary emphysema, hepatic cirrhosis, arthritis rheumatoid, and various types of cancer. Also in organ transplants and diseases due to radiation, smoking, and pollution and in the aging process, ROS play an important role. The results published in this special issue have shown that the participation of DNA damage in aging is a topic that involves many different factors, which are related to each other forming a metabolic network that is being unveiled and that opens a range for new investigations.

*Sharbel Weidner Maluf* *Wilner Martínez-López* *Juliana da Silva*

